# Prognostic value and reproducibility of different microscopic characteristics in the WHO grading systems for pTa and pT1 urinary bladder urothelial carcinomas

**DOI:** 10.1186/s13000-019-0868-3

**Published:** 2019-08-14

**Authors:** Vebjørn Kvikstad, Ok Målfrid Mangrud, Einar Gudlaugsson, Ingvild Dalen, Hans Espeland, Jan P. A. Baak, Emiel A. M. Janssen

**Affiliations:** 1 Department of Pathology, Stavanger University Hospital, Stavanger, Norway; 2 Department of Mathematics and Natural Science, University of Stavanger, Stavanger, Norway; 3 Department of Pathology, Innlandet Hospital, Lillehammer, Norway; 4 Department of Research, Stavanger University Hospital, Stavanger, Norway; 5 Department of Urology, Stavanger University Hospital, Stavanger, Norway; 6 Medical practice Dr. med. Jan Baak AS, Tananger, Norway; 7 Department of TCM, Faculty of Sports and Health Sciences, Technical University Munich, Munich, Germany

**Keywords:** Papillary urothelial carcinoma, Grading, Reproducibility, Prognosis

## Abstract

**Background:**

European treatment guidelines for pTa and pT1 urinary bladder urothelial carcinoma depend highly on stage and WHO-grade. Both the WHO73 and the WHO04 grading systems show some intra- and interobserver variability. The current pilot study investigates which histopathological features are especially sensitive for this undesired lack of reproducibility and the influence on prognostic value.

**Methods:**

Thirty-eight cases of primary non-muscle invasive urothelial carcinomas, including thirteen cases with stage progression, were reviewed by three pathologists. Thirteen microscopic features were extracted from pathology textbooks and evaluated separately. Reproducibility was measured using Gwet’s agreement coefficients. Prognostic ability regarding progression was estimated by the area under curve (AUC) of the receiver operating characteristics (ROC) function.

**Results:**

The best reproducible features (Gwet’s agreement coefficient above 0.60) were papillary architecture, nuclear polarity, cellular maturation, nuclear enlargement and giant nuclei. Nucleoli was the strongest prognostic feature, and the only feature with an AUC above 0.70 for both grading systems, but reproducibility was not among the strongest. Nuclear polarity also had prognostic value with an AUC of 0.70 and 0.67 for the WHO73 and WHO04, respectively. The other features did not have significant prognostic value.

**Conclusions:**

The reproducibility of the histopathological features of the different WHO grading systems varied considerably. Of all the features evaluated, only nuclear polarity was both prognostic and significantly reproducible. Further validation studies are needed on these features to improve grading of urothelial carcinomas.

## Background

Bladder cancer is the ninth most frequently diagnosed cancer worldwide. The incidence is highest in developed countries, and is the fourth most common cancer among men in Norway [1, 2]. Urothelial carcinoma accounts for about 90% of bladder cancers in industrialized countries [3], and 70–80% of these are non-muscle-invasive bladder cancers (NMIBC), pTa, pT1 or pTis, on first diagnosis. Among these 50–70% will recur, while only 15–25% will progress to a higher stage [4]. The follow-up of these patients is labor-intensive [5, 6], causing massive costs for the health care systems [7].

Papillary urothelial carcinomas are the most frequent in western countries and are graded based on the degree of anaplasia. In 1973 the World Health Organization (WHO) introduced a classification system, in which papillary carcinomas were divided into three groups; grades 1, 2 and 3 (WHO73). A new classification system was introduced in the 2004 WHO Classification of tumours of the urinary system (“blue book”), following an International Society of Urological Pathology (ISUP) consensus conference in 1998 (WHO04). This grading system is maintained in the 4th.edition, 2016, of the WHO blue book. Currently, both systems are being used in routine diagnostics at pathology departments around the world [8]. The WHO04 classification system divided the papillary urothelial tumours into papillary urothelial neoplasm of low malignant potential (PUNLMP), low and high grade carcinomas. The histologic features are described in detail, aiming to improve reproducibility. However, several studies have shown considerable interobserver variability for both classification systems [9–11]. In a recent review Soukup et al. [12] conclude, on behalf of the European Association of Urology (EAU), that the “Current grading classifications in NMIBC are suboptimal”, both with regards to reproducibility (poor to fair) and with regards to prognostication.

Grading of papillary urothelial carcinomas according to the WHO73 and the WHO04 classification systems is based on a variety of histopathological features. However, these are not necessarily consciously and systematically analysed one-by-one in a routine diagnostic setting by diagnostic pathologists. Rather than a time consuming analytical approach, many pathologists make a first-glance low-magnification diagnosis, and zoom in on special areas or features to get their diagnosis confirmed. This is a quick, time-effective method but a drawback is lack of reproducibility, with classification shifts from one to other grades and hence prognostic variation as well.

The aim of this pilot study was to systematically analyse the reproducibility and prognostic value of each of the microscopic features. As far as we know, this has not been done before; although previous work on mitotic activity in urothelial carcinoma has found mitosis to be a prognostic factor [13, 14].

## Methods

The study was approved by the Norwegian Regional Ethics Committee (#106/09). All patients with a primary non-muscle-invasive papillary urothelial carcinoma, at Stavanger University Hospital (SUH) from January 2002 to January 2007 were investigated (*N* = 228). All patients with urothelial carcinoma outside the urinary bladder (except for those with tumour in the pericollicular area in the urethra) were excluded. Thirty-five cases were excluded because of inadequate sample quality (necrotic tumour, fragmentation, thermal damage and insufficient material), leaving a total of 185 patients. Of these, 13 patients had stage progression; 12 within 5 years, and one after 5 years and 1 month.

In this pilot study we selected a group of 38 patients, including the 13 with progression and 25 without progression. Among the 13 patients with progression 10 were high grade and 3 were low grade according to WHO04. Patients without progression were randomly selected from the remaining 172 patients. There were no statistical significant differences between the grade, age, sex, recurrence or follow-up time of the selected 25 and the other 147 patients without progression.

Tumour tissue was obtained by transurethral resection or biopsy. Tissue was fixed in 4% buffered formaldehyde, dehydrated and embedded in paraffin. For microscopic evaluation four μm thick sections stained with haematoxylin-eosin-saffron (HES) were used.

The patients were treated according to the national guidelines at the time of diagnoses. The treatment consisted of transurethral resection (TUR), followed by a single instillation of a cytotoxic agent (epirubicin hydrochloride). Most patients defined as high risk patients were offered regular instillations with Bacillus Calmette Guérin (BCG), but some were offered alternative treatment with regular instillations containing a combination of epirubicin hydrochloride and interferon alpha. High risk patients included stage T1, grade 3 (WHO73), concurrent or later carcinoma in situ (pTis), three or more separate tumours diagnosed within 18 months or recurrences at multiple sites at first or second follow-up. Provided that the first follow-up cystoscopy was negative, patients with Ta grade 1 tumours would undergo control cystoscopies 3 months after initial diagnosis, 9 months later, and then annually for 5 years. All other patients would have cystoscopies every 3 months for the first 2 years, every 4 months for the 3rd year, every 6 month the 4th and 5th years, followed by annual cystoscopies thereafter.

Follow-up data were retrieved from the medical- and laboratory records at SUH. We defined progression as any advance in TNM stage, including both from pTa to pT1 or to pT2, and from pT1 to pT2. Progression to muscle invasive disease is clinically most relevant due to major differences in therapy. We also included cases with progression from pTa to pT1 as these tumours have gained the capability to infiltrate the stroma, a basic trait for progression.

The histopathological features constituting the grading systems were derived from urological pathology textbooks [15–17]. A list of the microscopic features and their interpretation, both for WHO73 and WHO04, is shown in Table 1. We extracted 13 features: papillae architecture, superficial layer, papillary fusion, nuclear polarity, cell maturation, cohesion, mitoses, nuclear enlargement, nuclear shape, nuclear hyperchromasia, chromatin pattern, nucleoli and giant nuclei.

**Table 1:** The microscopic features with descriptions for each grade (WHO73/ 04)

	WHO73			WHO04	
	Grade 1	Grade 2	Grade 3	Low grade	High grade
Architecture					
Papillae	Delicate	Varies	Broad, varies	Slender	Broad
Superficial layer (umbrella cell layer)	Usually present	Usually present	Partially or completely lost	Usually present	Partially or completely lost
Papillary fusion	Some	Varies	Common	Some	Varies
Nuclear arrangement					
Polarity	Preserved	Moderate loss	Lost	Preserved, moderate loss	Lost
Maturation	Normal	Some	Lost	Preserved, moderate loss	Lost
Cohesion	Normal	Some	Lost	Some	Lost
Proliferation					
Mitotic figures	Rare, basal	Lower half	Common, atypical	Rare	Common
Nuclear atypia					
Nuclear enlargement	Mild	Mild	Varies	Mild	Varies
Nuclear shape	Uniform	Moderate variation	Pleomorphic	Moderate variation	Pleomorphic
Nuclear hyperchromasia	Mild	Moderate	Varies	Mild to moderate	Varies
Chromatin pattern	Finely granular	Granular	Coarse	Fine	Coarse
Nucleoli	Occasional	Occasional	Common	Occasional	Common
Giant nuclei	No	No	Yes	No	Yes

All specimens were evaluated by three pathologists, focusing on grading criteria of the individual features, one at a time, for both WHO73 and WHO04. In tumours with morphological heterogeneity the “worst” area was graded. The evaluations were done without any knowledge about the original diagnosis or the other pathologists’ results. At a later stage, all three pathologists contributed to a consensus assessment for all the variables. Concerning the WHO04, only low grade and high grade were used as only three cases were classified as PUNLMP in our original cohort. In a previous study we found that recurrence and stage progression in the PUNLMPs and the low grade tumours by univariate survival analysis on our material were no different [18]. A later publication by Kim et al. [19] also showed no difference in progression between PUNLMP and low grade carcinomas.

### Statistics

Reproducibility was measured using Gwet’s AC_1_ agreement coefficient [20] for features with two categories, and using Gwet’s AC_2_ agreement coefficient with quadratic weights for features with > 2 categories [21]. Fleiss’ generalized kappa [22] is also reported for reference; however, due to its vulnerability to skewed marginal distributions [23], the focus in this paper is on Gwet’s agreement coefficients. A coefficient of < 0.2 is defined as poor agreement, 0.2–0.4 fair agreement, 0.4–0.6 moderate agreement, 0.6–0.8 good agreement and > 0.8 as very good agreement [24]. Confidence intervals (CIs) for the reliability measures were based on the normal approximation [21].

Prognostic ability with regard to progression for the consensus classification of each feature was estimated by the area under curve (AUC) of the receiver operating characteristics (ROC) function, which is reported with a normal based confidence interval [25]. Statistical analysis was performed in R version 3.4.0 with syntax provided at http://www.agreestat.com/r_functions.html (downloaded 24.05.2018) and with package pROC [25].

## Results

The median age at diagnosis was 72 years (range 56–87). Thirty patients were male (79%) and eight female (21%) (M:F ratio = 3.8). Median follow-up time was 73 months (range 5–168). Not all samples were regarded adequate for assessing all the microscopic features by all three pathologists. These cases were not included in the calculation of reliability for that particular feature (Table 2). At the consensus meeting, there was agreement that two cases could not be used to assess the feature “papillary fusion”. There were also two cases in which “maturation” could not be reliably assessed, and in one case “superficial layer” could not be assessed. This left between 36 to 38 total cases for each of the different features.

**Table 2:** Reproducibility and prognostic value for each of the microscopic characteristics

Feature	n*	AC_1_/AC_2_ (95% CI)	Fleiss’ κ (95% CI)	n**	Consensus grade (prob. of progression)	AUC_ROC_ (95% CI) ***
Papillae73	36	0.62 (0.42 to 0.82)	0.63 (0.45 to 0.82)	38	Delicate (1/10) Varies (4/11) Broad, varies (8/17)	0.67 (0.51 to 0.83)
Papilae04	36	0.61 (0.39 to 0.82)	0.59 (0.37 to 0.81)	38	Slender (3/16) Broad (10/22)	0.64 (0.49 to 0.80)
Superficial layer73/04	36	0.51 (0.30 to 0.73)	0.50 (0.29 to 0.72)	37	Usually present (4/12) Partially lost (8/25)	0.49 (0.33 to 0.66)
Papillary fusion73	34	0.64 (0.44 to 0.84)	0.67 (0.48 to 0.86)	36	Some (2/13) Varies (4/9) Common (7/14)	0.67 (0.50 to 0.84)
Papillary fusion04	34	0.53 (0.32 to 0.75)	0.53 (0.31 to 0.75)	36	Some (4/19) Varies (8/17)	0.67 (0.51 to 0.84)
Polarity73	38	0.68 (0.53 to 0.82)	0.70 (0.55 to 0.84)	38	Preserved (1/9) Moderate (4/14) Lost (8/15)	0.70 (0.54 to 0.86)
Polarity04	38	0.66 (0.47 to 0.86)	0.63 (0.43 to 0.84)	38	Preserved (5/23) Lost (8/15)	0.67 (0.50 to 0.83)
Maturation73	36	0.60 (0.43 to 0.78)	0.59 (0.42 to 0.76)	36	Normal (1/9) Some (5/14) Lost (6/13)	0.66 (0.49 to 0.83)
Maturation04	36	0.62 (0.42 to 0.82)	0.60 (0.40 to 0.81)	36	Some (6/23) Lost (6/13)	0.60 (0.43 to 0.78)
Cohesion73	37	0.57 (0.42 to 0.71)	0.47 (0.28 to 0.65)	38	Normal (1/12) Some (9/21) Lost (3/5)	0.71 (0.56 to 0.85)
Cohesion04	37	0.54 (0.30 to 0.77)	0.23 (−0.02 to 0.47)	38	Some (10/33) Lost (3/5)	0.58 (0.44 to 0.71)
Mitosis73	38	0.47 (0.23 to 0.71)	0.41 (0.18 to 0.64)	38	Rare, basal (8/31) Lower half (1/1) Common, atypical (4/6)	0.65 (0.50 to 0.80)
Mitosis04	38	0.64 (0.43 to 0.85)	0.49 (0.25 to 0.72)	38	Rare (9/32) Common (4/6)	0.61 (0.47 to 0.76)
Nuclear enlargement73/04	38	0.65 (0.45 to 0.85)	0.65 (0.45 to 0.84)	38	Mild (4/19) Varies (9/19)	0.65 (0.48 to 0.81)
Nuclear shape73	38	0.58 (0.41 to 0.74)	0.51 (0.32 to 0.69)	38	Uniform (3/10) Moderate (8/23) Pleomorphic (2/5)	0.53 (0.36 to 0.71)
Nuclear shape04	38	0.58 (0.34 to 0.81)	0.41 (0.21 to 0.61)	38	Moderate (11/33) Pleomorphic (2/5)	0.52 (0.40 to 0.64)
Nuclear hyperchromasia73	38	0.51 (0.38 to 0.65)	0.51 (0.35 to 0.68)	38	Mild (3/11) Moderate (6/17) Varies (4/10)	0.56 (0.37 to 0.74)
Nuclear hyperchromasia04	38	0.51 (0.28 to 0.74)	0.43 (0.21 to 0.65)	38	Mild to moderate (9/28) Varies (4/10)	0.53 (0.38 to 0.69)
Chromatin pattern73	38	0.51 (0.29 to 0.73)	0.46 (0.26 to 0.67)	38	Finely granular (7/25) Granular (4/10) Coarse (2/3)	0.60 (0.43 to 0.78)
Chromatin pattern04	38	0.66 (0.47 to 0.86)	0.55 (0.31 to 0.79)	38	Fine (9/31) Coarse (4/7)	0.59 (0.45 to 0.74)
Nucleoli73/04	38	0.54 (0.33 to 0.76)	0.54 (0.33 to 0.75)	38	Occasional (2/16) Common (11/22)	**0.70 (0.56 to 0.85)**
Giant nuclei	38	**0.85 (0.72 to 0.98)**	0.78 (0.59 to 0.98)	38	No (8/28) Yes (5/10)	0.59 (0.43 to 0.75)

*AC*_*1*_*/ AC*_*2*_ Gwet’s AC_1_/ AC_2_ coefficient, *CI* Confidence interval, *AUC*_*ROC*_ Area under Receiver Operating Characteristics Curve.

* Number of cases evaluated by all three pathologists

** Number of cases for which consensus was reached

The reproducibility varies among the different microscopic features according to the calculated Gwet’s AC_1/2_ agreement coefficient (Table 2). The values range from 0.47 for mitosis in the WHO73 system to 0.85 for giant nuclei. This corresponds to moderate to very good reproducibility. The other features yielded evenly distributed values, with papillae architecture, nuclear polarity, cell maturation, nuclear enlargement and giant nuclei as the most reproducible, all with Gwet’s AC_1/2_ agreement coefficient above 0.60 (=good agreement) for both grading systems. Several of the values have very wide confidence intervals, making them less robust. For instance, for mitosis73 the confidence interval ranges from 0.23 to 0.71.

Prognostic ability for the different features, estimated by AUC, ranged from 0.49 for superficial layer, to 0.71 for cohesion in WHO73. To qualify as reliable, we wanted the features to be convincing (> 0.7) for both WHO73 and WHO04. For instance, cohesion generated an AUC of 0.58 for WHO04, and should therefore not be relied on in our material. Only nucleoli achieved an AUC above 0.7 for both WHO73 and WHO04, which is seen as an acceptable discrimination for progression or not. Polarity tends to show some prognostic information for both grading systems with AUC 0.70/ 0.67 for WHO73 and WHO04 respectively. These two features and papillary fusion gave estimated confidence intervals ≥0.5 for both grading systems. The other ten features showed no statistical significant prognostic value.

Nuclear polarity was the only feature with both reasonable reproducibility and prognostic value in this pilot study.

## Discussion

Grade is seen as one of the most important prognostic factors in bladder cancer, with impact on treatment and patient follow-up. As reproducibility of both WHO73 and WHO04 is suboptimal, we systematically analysed the reproducibility and prognostic value of each of the microscopic features described as being part of grading. Each of the 13 features, which theoretically should be used to reach the final grade, carries its own uncertainty in terms of reproducibility and prognostic value.

In the absence of a formal prognostic decision tree of microscopic features in urinary bladder cancers, and lack of a descriptive atlas with typical pictures, pathologists will emphasize each feature differently while grading a urinary bladder tumour. The assessment of grade is therefore more or less based on intuition, as the features are not evaluated in a systematic manner, and only rarely truly quantitatively. This partially explains the considerable difficulty with reproducibility. Furthermore, the thresholds for the different subclasses of each of the included features are very subjective (example: the described thresholds for cohesion are: normal, some or lost). Such descriptive and subjective criteria lead to diagnostic confusion. In the process of grading, pathologists will also be challenged by laboratory variables like section thickness which might blur nuclear hyperchromasia or the introduction of artefacts that might mimic dyscohesiveness. The individual prognostic values of these features has never been analysed separately in urinary bladder tumours.

Before our analyses we expected mitoses to be a useful feature, as reported in a previous study on bladder cancer [13]. In the current analyses, mitosis was one of the least reproducible and prognostic features. However, mitotic activity in the current study was assessed in a semi-quantitative manner. Contrary, previous studies which reported mitoses as a strong prognostic factor, counted mitoses in a defined area by using the protocol for Mitotic Activity Index (MAI) as it is used and developed for breast cancer, and the final number of mitoses was used to categorize the tumours. When grading according to either of the WHO-systems, a rough mitotic impression, rather than a formalized mitotic count is used. This may explain the differences in prognostic value and reproducibility. Such a prognostic difference between mitotic activity as the MAI (truly quantitative) and mitotic impression (a rough estimate) has previously been shown in breast cancer [26], and may be true for urothelial carcinoma as well.

To be clinically useful, a grading system should be well reproducible to assure the intended sensitivity and specificity. As known the final grade is the sum of an evaluation of different microscopic features, therefore if one of these features is not truly quantitative, it inevitably will lack reproducibility and this will affect the final grade as well. Individual features may have a prognostic potential, which might be hidden by low overall reproducibility. It is crucial to minimalize the interobserver variability, making these features more reliable before extracting and emphasizing the features giving the best prognostic information. These features might be evaluated separately in a new grading system.

One way to improve reproducibility could be to provide pathologists with an image atlas with examples of the various features, facilitating comparison with the tumour to be graded. In prostate adenocarcinoma, the Gleason score has been well documented, tested and tried since its introduction in 1966 [27]. It has been claimed that the success of the system may in part be attributed to the ease of application and the simplicity of the original drawings [15]. Although the Gleason score has issues regarding reproducibility as well, especially when differentiating between Gleason grade group 2 and 3 [28, 29], the system as a whole has proven to be an important predictor of prognosis [30, 31]. A similar system with simplified, stylized illustrations may improve grading reproducibility in bladder cancer as well.

**Fig. 1:**
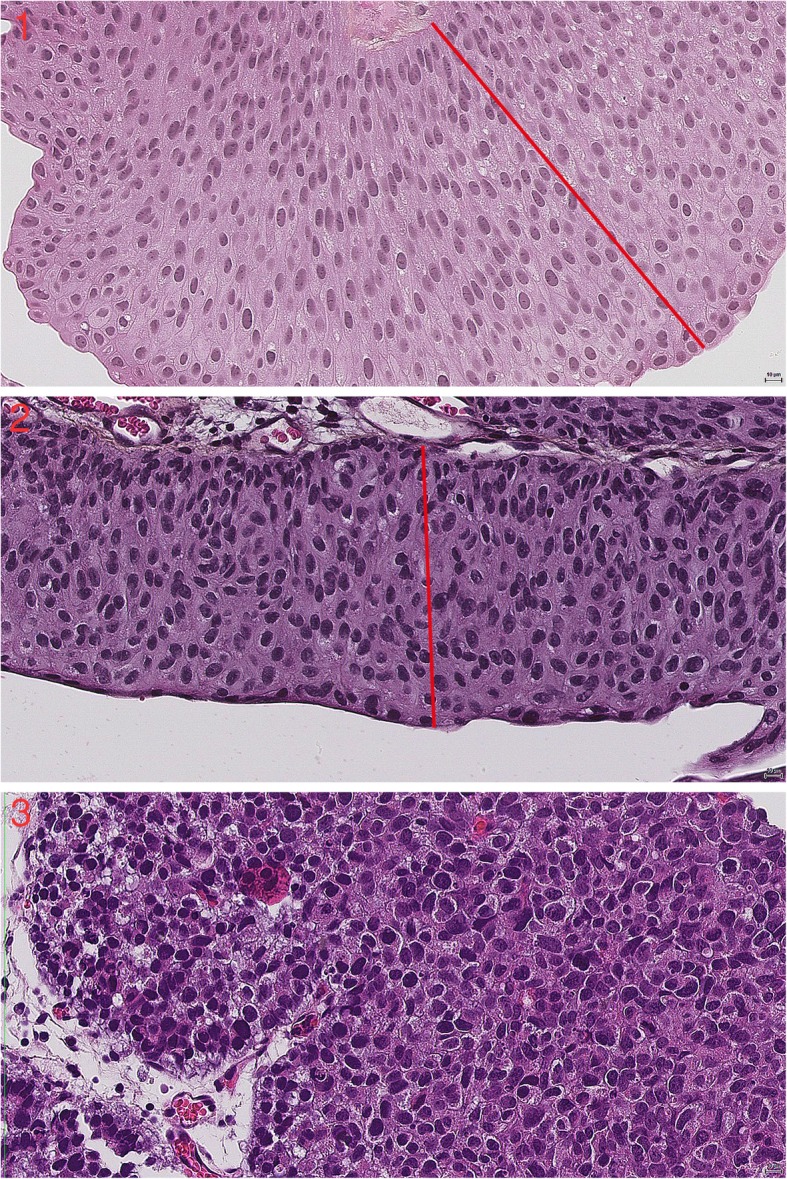
The images 1–3 show decreasing nuclear polarity at 40 x magnification. The red line is for comparison with the axis of the nuclei

In this study nuclear polarity stands out as the most valuable histopathological feature in grading. This supports the current view that architectural and cytological order versus disorder decides whether a lesion should be regarded as low or high grade in the WHO04 grading system. Strict definitions will be necessary to further improve reproducibility of this feature as well. One approach could be to grade nuclear polarity according to how much the axis of the nuclei tends to deviate from a line perpendicular to the basement membrane (Fig. 1).

The introduction of digital pathology introduces a multitude of possibilities for measurement of structures like nuclei, nucleoli and papillae. This can be exploited in grading, in an attempt to achieve standardization. Digital images can be further analysed by computer based algorithms, thereby analysing features not easily measured directly, like polarity, nuclear shape and mitotic Figures. A first attempt, using a local binary pattern (LBP) and local variance (VAR) operators followed by a RUSboost classifier, on a small test set of 42 patients with NMIBC resulted in an accuracy of 70%, a sensitivity of 84% and a specificity of 45% for prediction of recurrences [32]. Although only performed using a small dataset these results show the potential of these methods. Further studies using bigger datasets are necessary to further investigate these new measurements.

The value of the data in this pilot study is limited by the small sample size, not allowing any final conclusions. Although, our data suggest a substantial variety among the different histopathological features when it comes to reproducibility. Also, the prognostic value is disappointing for most of the features. Our data calls for further validation studies to highlight the most reproducible and most prognostic microscopic features making up the current grading system. We hope this article will contribute to developing a new approach.

when it comes to grading of papillary urothelial carcinomas.

## Conclusion

WHO grading is based on the use of 13 histopathological features, which in our material vary considerably in reproducibility and prognostic value. Of all the features evaluated in this small study, only nuclear polarity was both reasonably prognostic and reproducible. Further validation studies on the individual histopathological features are needed to improve the assessment of grade of urothelial carcinomas. A new grading system should be based upon more clear-cut definitions and features with true prognostic value.

## Data Availability

The datasets used and analysed during the current study are available from the corresponding author on reasonable request.

## Abbreviations

AUC: Area under curve
BCG: Bacillus Calmette Guérin
CI: Confidence interval
EAU: European association of urology
HES: Haematoxylin-eosin-saffron
ISUP: International Society of Urological Pathology
LBP: Local binary pattern
MAI: Mitotic Activity Index
NMIBC: Non-muscle-invasive bladder cancer
PUNLMP: Papillary urothelial neoplasia of low malignant potential
ROC: Receiver operating characteristics
SUH: Stavanger University Hospital
TUR: Transurethral resection
VAR: Local variance
WHO: World Health Organization
WHO04: The World Health Organization grading system from 2004
WHO73: The World Health Organization grading system from 1973
